# Temporal series analysis of abiotic data for a subtropical Brazilian rocky shore

**DOI:** 10.1016/j.dib.2019.103873

**Published:** 2019-03-25

**Authors:** Marcos Gonçalves da Silva, Juliana Nascimento Silva, Helena Rodrigues Fragoso, Natalia Pirani Ghilardi-Lopes

**Affiliations:** Federal University of ABC - UFABC Rua Arcturus, 03, Jardim Antares, São Bernardo do Campo, São Paulo, CEP 09606-070, Brazil

**Keywords:** Temporal series analysis, Ocean-atmospheric data, Rocky shore communities, SST, Sea Surface Temperature, SSS, Sea Surface Salinity, PAR, Photosynthetically Available Radiation

## Abstract

Monthly data of abiotic variables (sea surface temperature; minimum and maximum air temperature; minimum, mean and maximum air humidity; minimum, mean and maximum atmospheric pressure; minimum, mean and maximum dew point; sea surface salinity; wind speed and direction; minimum and maximum tidal level and photosynthetically available radiation) were collected from different online repositories, all regarding the period between January 2013 and December 2017, from localities near Mar Casado Beach rocky shore, in São Paulo State southern coast, Brazil. Principal Component Analysis was performed to verify data variance and correlations among variables. Linear regression decomposition methods were applied to identify trend and seasonal patterns within the time indexed data. Deseasonalized time series were analyzed to identify structural breaks in trend patterns. Spectral analysis was applied to detrended time series.

**Table:** Specifications table

Subject area	*Biology*
More specific subject area	*Marine ecology*
Type of data	*Table, figures*
How data was acquired	*Data collected from online open access repositories*
Data format	*Filtered and analyzed*
Experimental factors	*Before analyses were performed, the sampled months of January to March (Summer); April to June (Autumn); July to September (Winter) and October to December (Spring) were averaged.*
Experimental features	*Co-variant abiotic factors were identified through principal component analysis. Trend and seasonal patterns were extracted from collected data using time series analysis methods. Structural breaks in trend pattern and seasonal periodicity were also analyzed.*
Data source location	*Southeast coast of Brazil (São Paulo State southern coast)*
Data accessibility	*All data are presented in this article*

**Table:** 

**Value of the data** • These data describe variations in abiotic factors from 2013 to 2017 at São Paulo State southern coast. It may be used in studies that correlate them to the temporal dynamics of marine communities, in order to understand biotic responses to environmental disturbances; • This dataset may be used as a baseline for long-term monitoring databases of abiotic variables in coastal Brazilian areas; • The data may be used in comparative analysis of the temporal dynamics of abiotic data among different sites.

## 1 Data

Intertidal rocky shore communities are daily and seasonally exposed to a variety of abiotic stressors, such as variations in tidal level, air and sea water temperature, solar radiation and desiccation rates. These abiotic factors are known to affect survival and reproduction of organisms inhabiting the intertidal zone of rocky shores [1]. Although there are monitoring initiatives of marine communities for the Brazilian coast (e.g., ReBentos monitoring sites [2]), the monitoring of abiotic factors is still scarce. In an attempt to supplant this lack, we gathered historical data available in different online databases, all concerning Brazilian southeast coast, specifically São Paulo State southern coast (Table 1), from localities near Mar Casado Beach rocky shore. Thus, biotic responses to environmental factors may be studied in a temporal context.

**Table 1:** Monthly data for 18 abiotic variables, from January 2013 to December 2017. All data were collected from public repositories available online, regarding multiple sites within the southeast coast of Brazil, specifically at São Paulo State southern coast.

	SST (ºC)	Max temperature (ºC)	Min temperature (ºC)	Max humidity (%)	Min humidity (%)	Mean humidity (%)
jan/13	26,27	27,94	20,84	95,84	63,87	79,86
feb/13	26,27	31,11	22,00	97,07	57,61	77,34
mar/13	26,57	28,35	21,48	95,68	63,81	79,75
apr/13	24,82	26,77	18,20	98,6	59,90	79,25
may/13	24,05	25,81	17,16	95,19	57,61	76,40
jun/13	22,37	24,03	17,17	96,40	66,90	81,65
jul/13	20,73	22,1	13,94	94,39	64,29	79,34
aug/13	18,85	23,39	14,29	92,10	58,19	75,15
sep/13	21,05	24,30	16,40	92,63	60,77	76,70
oct/13	21,79	24,87	18,26	92,26	63,50	77,88
nov/13	22,89	26,73	20,33	90,83	63,53	77,18
dec/13	25,30	28,90	21,61	91,87	60,48	76,18
jan/14	28,73	32,55	22,71	91,48	54,03	72,76
feb/14	28,09	31,29	22,82	91,39	56,32	73,86
mar/14	27,10	29,68	21,65	94,29	62,19	78,24
apr/14	26,11	27,27	19,50	95,60	62,33	78,97
may/14	23,89	25,58	17,35	96,52	61,00	78,76
jun/14	22,20	25,00	16,73	98,63	62,07	80,35
jul/14	21,60	23,58	15,29	98,32	60,45	79,39
aug/14	21,63	24,94	15,13	98,68	57,48	78,08
sep/14	22,09	25,37	17,67	97,9	61,87	79,89
oct/14	22,96	26,52	18,65	96,32	57,42	76,87
nov/14	23,70	27,20	20,30	98,63	61,70	80,17
dec/14	24,86	28,65	22,00	95,42	59,65	77,54
jan/15	28,68	31,52	24,48	94,06	59,90	76,98
feb/15	28,47	29,68	22,86	98,39	66,57	82,48
mar/15	27,27	28,77	24,03	94,97	69,06	82,02
apr/15	25,12	27,2	20,77	99,2	65,23	82,22
may/15	23,91	26,00	17,00	97,00	65,90	81,45
jun/15	23,27	24,00	17,00	99,00	54,60	76,80
jul/15	21,81	24,90	16,68	89,03	61,03	75,03
aug/15	22,51	26,26	16,29	90,81	56,13	73,47
sep/15	22,49	25,37	18,77	90,73	64,63	77,68
oct/15	23,24	24,84	20,19	90,45	68,90	79,68
nov/15	24,65	26,23	21,57	91,97	71,03	81,50
dec/15	25,52	28,65	22,87	91,00	63,71	77,36
jan/16	25,22	28,90	22,10	95,97	66,29	81,13
feb/16	28,20	31,41	23,62	91,00	59,34	75,17
mar/16	27,24	29,61	22,61	96,19	67,55	81,87
apr/16	28,02	28,30	20,80	95,67	64,07	79,87
may/16	23,35	24,10	18,32	97,39	69,82	83,61
jun/16	19,06	19,33	14,27	99	77,1	88,05
jul/16	19,12	22,68	14,94	98,65	64,97	81,81
aug/16	19,58	23,13	15,35	97,87	70,39	84,13
sep/16	21,38	23,30	17,57	96,80	69,73	83,27
oct/16	22,66	25,45	19,74	95,97	71,77	83,87
nov/16	24,05	25,97	19,87	97,80	70,93	84,37
dec/16	26,16	29,35	21,71	95,48	64,03	79,76
jan/17	28,64	30,06	23,65	97	69,52	83,26
feb/17	28,07	30,82	22,82	95,11	65,14	80,13
mar/17	26,67	28,81	22,10	96,58	68,39	82,49
apr/17	25,44	26,47	20,23	98,20	69,63	83,92
may/17	22,54	25,03	18,81	90,13	63,03	76,58
jun/17	21,82	24,97	15,90	86,97	50,40	68,69
jul/17	21,61	23,68	13,94	86,58	48,90	67,74
aug/17	20,64	23,52	16,1	86,48	54,42	70,45
sep/17	22,00	25,60	18,10	84,50	56,50	70,50
oct/17	22,99	27,19	20,19	91,26	61,90	76,58
nov/17	22,65	26,60	19,13	95,80	64,40	80,10
dec/17	22,65	27,74	22,16	95,23	71,06	83,15

	Max pressure (Hg mm)	Min pressure (Hg mm)	Mean pressure (Hg mm)	Wind speed (km/h)	Wind direction (º)	Max tide (m)
jan/13	1013,35	1010,45	1012,10	5,47	51,80	1,25
feb/13	1013,61	1010,04	1011,89	3,83	71,58	1,27
mar/13	1015,35	1011,84	1013,48	6,22	54,58	1,30
apr/13	1017,27	1014,20	1015,67	5,37	70,64	1,28
may/13	1018,32	1014,55	1016,32	4,88	66,01	1,25
jun/13	1019,33	1015,60	1017,40	3,73	71,94	1,25
jul/13	1022,03	1018,58	1020,16	5,27	79,75	1,25
aug/13	1021,03	1016,87	1018,84	4,38	90,27	1,28
sep/13	1019,20	1015,03	1017,23	5,48	74,55	1,32
oct/13	1017,77	1013,84	1015,74	5,91	56,02	1,30
nov/13	1014,77	1010,97	1012,97	5,78	56,62	1,27
dec/13	1011,58	1008,45	1010,23	3,97	72,88	1,25
jan/14	1013,94	1010,81	1012,45	4,06	45,42	1,26
feb/14	1013,5	1010,39	1012,07	3,87	42,54	1,28
mar/14	1014,74	1011,97	1013,32	4,28	46,75	1,30
apr/14	1017,37	1014,17	1015,63	4,69	60,59	1,28
may/14	1018,35	1014,94	1016,48	5,14	79,75	1,27
jun/14	1019,67	1016,10	1017,87	4,30	68,80	1,25
jul/14	1023,16	1019,65	1021,35	6,95	54,38	1,26
aug/14	1021,03	1017,58	1019,42	5,24	60,67	1,29
sep/14	1018,8	1014,27	1016,4	4,65	69,59	1,31
oct/14	1018,65	1014,29	1016,48	5,15	52,22	1,30
nov/14	1014,60	1011,07	1012,93	5,10	50,26	1,27
dec/14	1013,42	1009,97	1011,84	3,06	63,66	1,23
jan/15	1013,90	1011,10	1012,71	3,01	72,92	1,25
feb/15	1012,71	1010,11	1011,61	4,51	60,84	1,27
mar/15	1014,55	1011,74	1013,16	4,68	57,67	1,27
apr/15	1016,9	1013,7	1015,2	6,64	58,47	1,28
may/15	1019,19	1015,84	1017,35	6,14	58,94	1,28
jun/15	1022,03	1018,17	1020,07	5,99	46,28	1,14
jul/15	1020,61	1016,32	1018,48	4,87	67,91	1,28
aug/15	1020,29	1016,13	1018,06	4,88	40,59	1,30
sep/15	1016,63	1012,03	1014,37	4,84	60,52	1,32
oct/15	1017,19	1013,23	1015,32	6,52	47,57	1,31
nov/15	1013,33	1010,03	1011,77	6,94	42,87	1,28
dec/15	1013,23	1009,94	1011,71	6,18	44,73	1,25
jan/16	1012,94	1009,77	1011,48	4,88	44,82	1,25
feb/16	1013,76	1010,69	1012,21	3,05	60,57	1,28
mar/16	1015,61	1012,65	1014,06	4,71	53,85	1,25
apr/16	1015,53	1012,50	1014,17	3,36	35,75	1,29
may/16	1020,16	1016,42	1018,26	7,08	66,32	1,27
jun/16	1023,23	1020,13	1021,63	4,47	115,28	1,25
jul/16	1021,55	1017,52	1019,45	4,08	67,71	1,27
aug/16	1020,48	1016,03	1018,06	4,63	74,42	1,28
sep/16	1019,79	1016,17	1017,97	5,57	78,91	1,32
oct/16	1016,45	1012,45	1014,48	8,53	37,90	1,33
nov/16	1015,03	1011,60	1013,43	6,89	61,50	1,29
dec/16	1013,10	1009,94	1011,65	6,76	78,49	1,27
jan/17	1012,97	1009,9	1011,55	6,25	64,42	1,26
feb/17	1013,48	1010,46	1012,07	5,27	60,12	1,27
mar/17	1015,26	1012,39	1013,81	5,58	70,69	1,30
apr/17	1017,77	1014,23	1015,87	7,80	44,63	1,28
may/17	1018,48	1015,10	1016,68	9,85	29,16	1,27
jun/17	1020,97	1017,50	1019,13	4,73	63,61	1,25
jul/17	1026,29	1022,77	1024,42	4,07	79,62	1,25
aug/17	1020,93	1016,45	1018,71	7,88	74,74	1,29
sep/17	1020,70	1017,10	1018,67	7,06	34,54	1,32
oct/17	1015,97	1011,26	1013,65	4,76	80,53	1,32
nov/17	1014,10	1010,20	1012,27	7,92	57,04	1,31
dec/17	1012,58	1009,45	1011,23	7,65	13,43	1,28

	Min tide (m)	Max dew point (ºC)	Min dew point (ºC)	Mean dew point (ºC)	PAR (E/m^2^s)	SSS (PSU)
jan/13	0,28	22,00	19,61	20,71	50,84	35,50
feb/13	0,26	23,39	20,25	22,11	51,48	35,13
mar/13	0,27	22,35	19,71	21,32	36,19	35,62
apr/13	0,29	20,40	16,93	18,80	35,78	35,67
may/13	0,26	18,55	15,19	17,23	27,58	35,77
jun/13	0,25	18,77	15,80	17,57	21,69	35,41
jul/13	0,29	16,19	12,42	14,9	25,69	34,45
aug/13	0,29	15,71	11,68	14,26	31,20	32,74
sep/13	0,29	17,33	13,90	15,73	33,23	32,32
oct/13	0,31	18,61	16,10	17,61	38,72	33,13
nov/13	0,30	20,10	17,16	18,42	46,23	35,22
dec/13	0,26	21,84	19,06	20,61	52,86	35,24
jan/14	0,24	23,71	20,19	22,19	60,23	35,45
feb/14	0,29	23,64	19,86	22,36	50,46	35,47
mar/14	0,25	23,06	20,19	21,84	43,70	35,36
apr/14	0,28	21,03	18,23	19,77	34,91	35,31
may/14	0,28	19,23	15,32	17,65	26,30	35,54
jun/14	0,26	19,03	15,27	17,47	22,50	35,80
jul/14	0,25	17,52	13,65	15,77	24,77	35,44
aug/14	0,28	18,00	13,32	15,77	31,83	35,45
sep/14	0,28	19,33	16,13	17,8	35,35	35,75
oct/14	0,29	19,68	16,16	17,94	43,65	35,70
nov/14	0,29	21,50	18,47	19,93	49,25	35,64
dec/14	0,28	22,45	19,06	20,90	50,30	35,79
jan/15	0,27	24,26	21,45	22,97	55,98	35,77
feb/15	0,29	24,29	21,57	22,79	49,11	35,36
mar/15	0,28	24,23	21,68	22,81	40,48	35,33
apr/15	0,28	22,07	19,3	20,73	34,49	35,62
may/15	0,34	18,70	16,80	17,80	26,85	35,54
jun/15	0,38	17,00	13,80	15,00	26,65	36,12
jul/15	0,25	17,84	14,19	16,06	23,88	36,56
aug/15	0,28	18,61	14,23	16,90	34,65	35,86
sep/15	0,27	19,30	16,27	17,93	32,15	35,68
oct/15	0,31	20,10	17,77	19,13	33,55	35,46
nov/15	0,30	21,3	19,43	20,33	37,79	34,19
dec/15	0,29	22,71	20,39	21,55	45,76	34,96
jan/16	0,28	23,06	20,55	21,81	46,37	35,35
feb/16	0,27	24,17	21,14	22,66	52,22	35,24
mar/16	0,28	24,10	21,32	22,94	44,12	35,42
apr/16	0,27	22,83	19,97	21,57	40,44	35,11
may/16	0,26	19,57	17,00	18,43	23,92	34,97
jun/16	0,26	16	13,5	14,97	21,06	31,93
jul/16	0,26	17,23	13,81	15,65	23,68	29,70
aug/16	0,25	18,00	14,55	16,71	28,22	31,74
sep/16	0,28	18,73	16,20	17,60	34,51	33,17
oct/16	0,29	20,87	18,87	19,87	42,72	32,77
nov/16	0,29	21,57	18,73	20,53	40,10	34,00
dec/16	0,27	23,06	20,16	21,81	55,24	34,24
jan/17	0,26	24,94	22,32	23,61	43,28	33,78
feb/17	0,25	24,68	21,46	23,25	53,57	35,07
mar/17	0,26	23,23	20,84	22,23	46,75	35,86
apr/17	0,30	21,80	19,13	20,67	30,74	35,51
may/17	0,28	18,77	16,13	17,65	29,20	36,62
jun/17	0,28	16,63	11,90	14,73	25,54	36,57
jul/17	0,28	15,32	10,32	13,06	30,88	36,44
aug/17	0,28	15,87	11,58	14,19	27,16	36,28
sep/17	0,26	17,60	14,47	16,53	42,48	36,14
oct/17	0,28	20,71	17,06	19,13	40,74	35,99
nov/17	0,29	20,50	17,47	19,20	46,95	35,98
dec/17	0,27	22,90	20,71	21,94	44,72	36,41

**Fig. 1:**
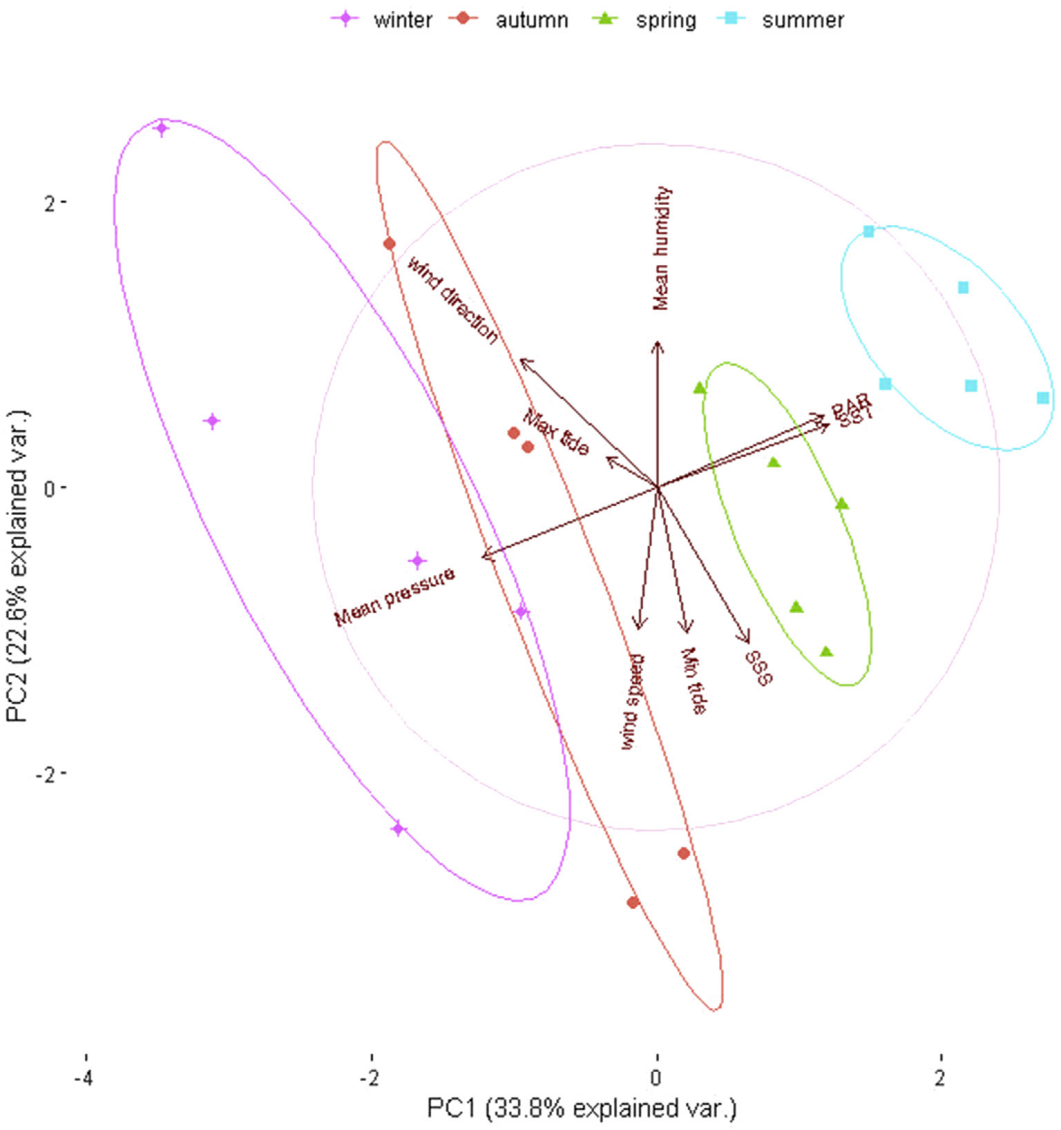
Principal component analysis after removal of co-variant abiotic variables, showing two distinct seasons: a cold and dry (purple and coral ellipses, encompassing Winter and Autumn, indicated by crosses and circles) and a hot and moist (green and blue ellipses, encompassing Spring and Summer, indicated by triangles and squares). SST: Sea Surface Temperature; SSS: Sea Surface Salinity; PAR: Photosynthetically Available Radiation; PC: Principal Component.

A principal component analysis of quarterly data (as explained further in section 2.2) recovered two distinct seasons: (I) a hot and moist season from October to March (encompassing Spring and Summer) and (II) a cold and dry season from April to September (encompassing Autumn and Winter); an expected result for subtropical zones [3] (Fig. 1). Principal Components (PC) 1 and 2 explain 56.4% of data variance. The plot shows only non-covariant variables identified after a first-round PCA considering all 18 variables, in which SST co-varied with maximum and minimum air temperature and also maximum, mean and minimum dew point. Mean atmospheric pressure co-varied with maximum and minimum values of this same abiotic variable. Also, mean air humidity co-varied with maximum and minimum values of this same abiotic variable.

**Fig. 2:**
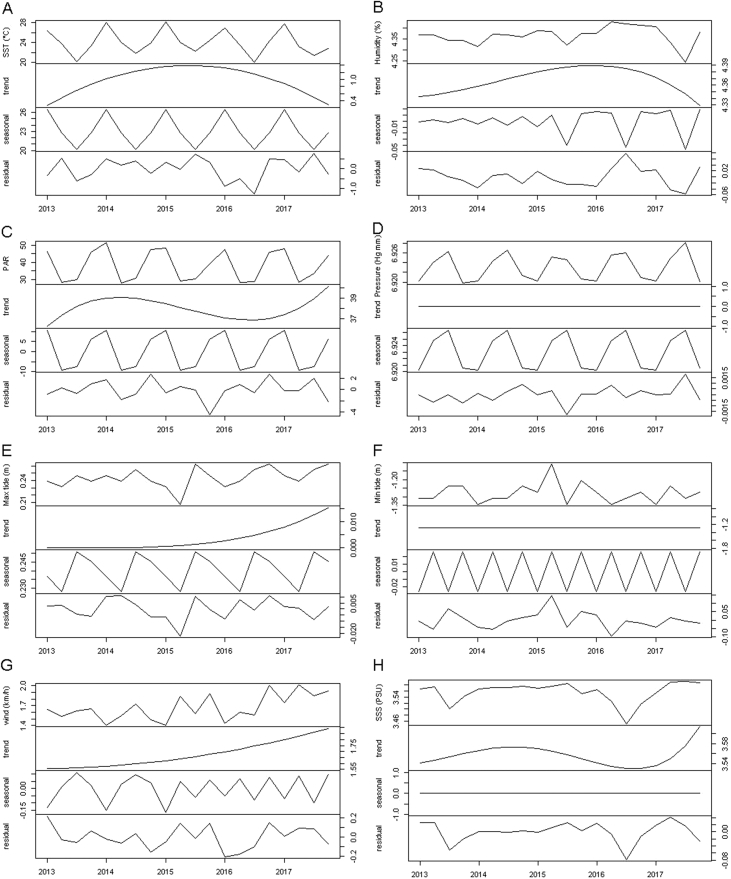
Time series decomposition. (A) Sea surface temperature (°C), additive decomposition applied to linear model with seasonal variables. (B) Mean air humidity (%), multiplicative decomposition applied to harmonic seasonal model, data log transformed. (C) Photosynthetically available radiation (E/m^2^s), additive decomposition applied to harmonic seasonal model. (D) Mean atmospheric pressure (Hg mm), multiplicative decomposition applied to linear model with seasonal variables, data log transformed. (E) Maximum tide level (m), multiplicative decomposition applied to linear model with seasonal variables, data log transformed. (F) Minimum tide level (m), multiplicative decomposition applied to harmonic seasonal model, data log transformed (G) Wind speed (Km/h), multiplicative decomposition applied to harmonic seasonal model, data log transformed. (H) Sea surface salinity, multiplicative decomposition applied to harmonic seasonal model, data log transformed.

Time series modelling was carried using only nine of the original 18 abiotic variables (SST, mean air humidity, PAR, mean atmospheric pressure, maximum and minimum tide level, wind speed, wind direction and SSS), as the remaining variables co-varied through time with some of the modelled data. Fig. 2 shows trend, seasonal variation and residuals decomposition for eight variables, since trend and seasonal features for wind direction time series could not be deterministically modelled, a result indicating that wind direction might be stochastic.

**Fig. 3:**
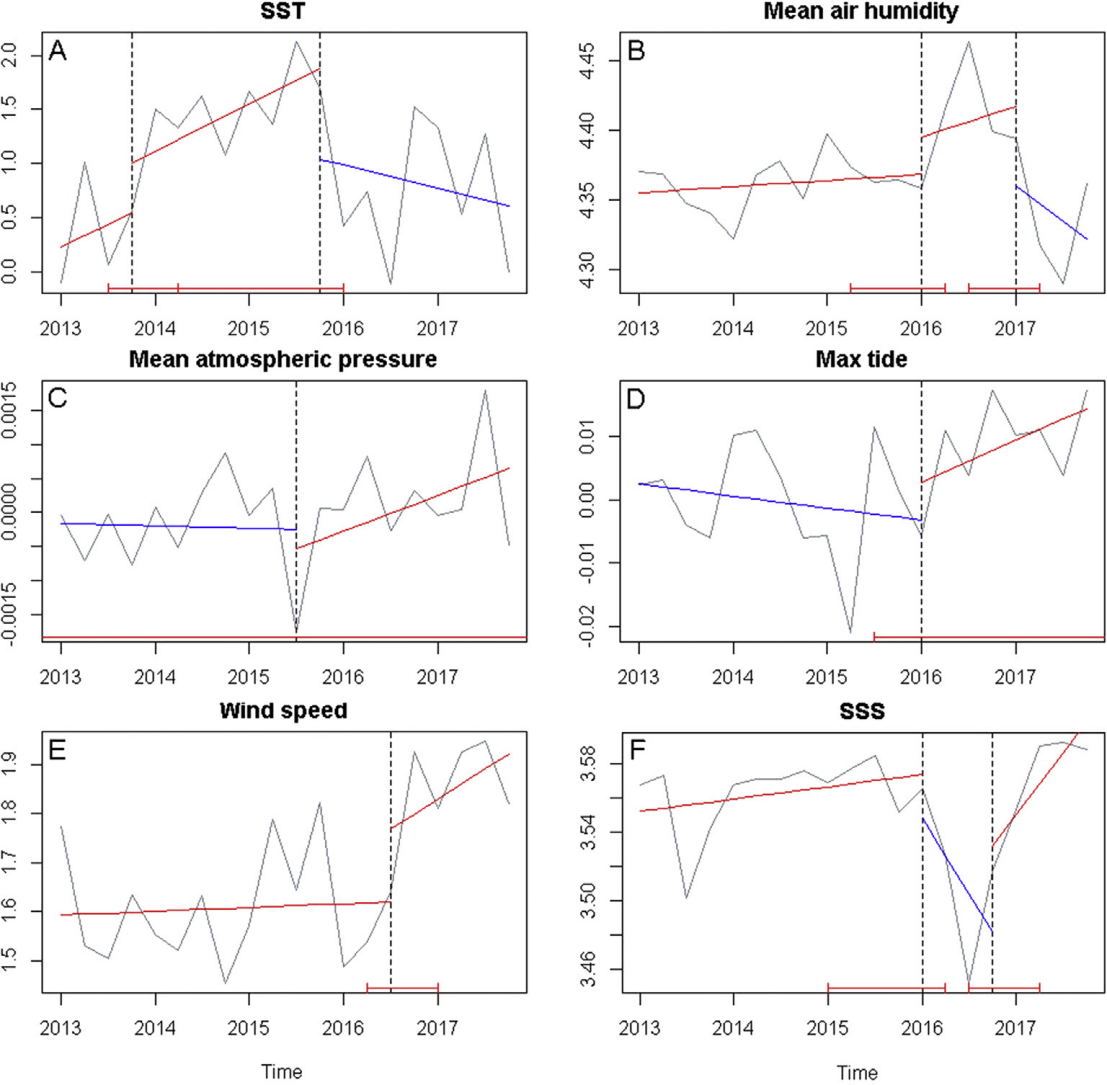
Structural break approach to trend analysis. Dashed vertical lines show breakpoints, bottom red bar shows the confidence interval and the trend line is plotted against deseasonalized time series, where red tilted up lines indicate an increasing trend and blue tilted down lines indicate a decreasing trend. (A) Sea surface temperature (°C), (B) mean air humidity (%), (C) mean atmospheric pressure (Hg mm), (D) maximum tide level (m), (E) wind speed (Km/h), (F) sea surface salinity (PSU).

Structural breaks in trend pattern were accessed through deseasonalized time series. These structural breaks are points in time in which statistical patterns change, highlighting sections of time when an increasing or a decreasing trend is more pronounced [4]. Fig. 3 shows deseasonalized time series, overlaid to their structural breaks (vertical lines) and increasing or decreasing trend for each time interval. No breakpoints were identified in minimum tide level time series, consistent with time series decomposition (Fig. 2F) in which no deterministic trend could be identified within data. No breakpoints were identified in PAR time series. While no deterministic trend could be identified in mean atmospheric pressure time series (Fig. 2D), a breakpoint could be identified (Fig. 3C) although the confidence interval stands outside the observation time interval. A confidence interval problem also occurred in maximum tide level structural break analysis (Fig. 3D). We expect that the number of breaks in a biotic variable time series (for example, the percentual cover of barnacles on the studied rocky shore) would be similar to those of a correlated abiotic variable.

**Fig. 4:**
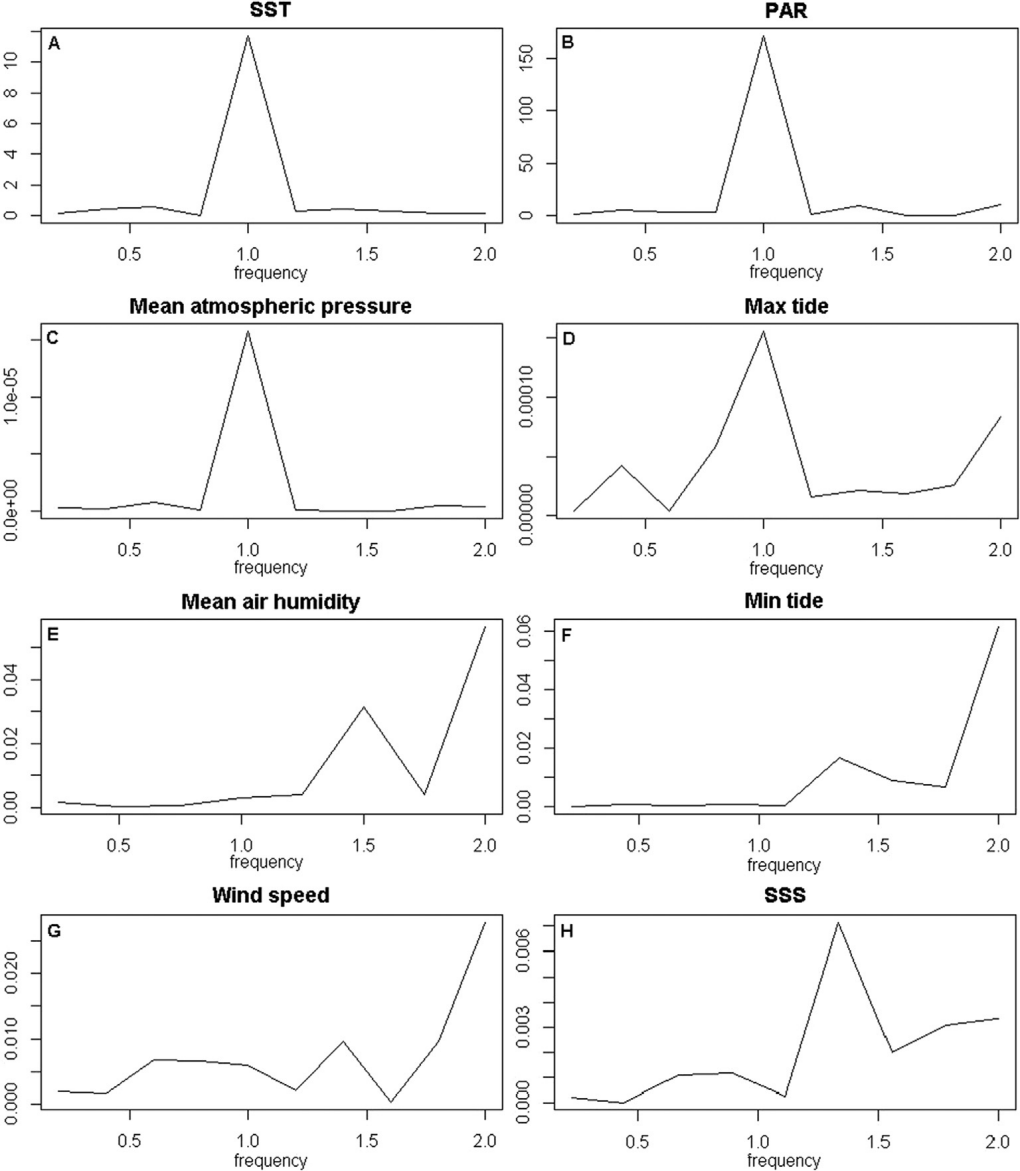
Spectral analysis. A dominant spike shows in which frequency seasonal events occur, where frequency 1.0 equals one sampling interval, or a quarterly rate. (A) Sea surface temperature (°C), (B) photosynthetically available radiation (E/m^2^s), (C) mean atmospheric pressure (Hg mm), (D) maximum tide level (m), (E) mean air humidity (%), (F) minimum tide level (m), (G) wind speed (Km/h) and (H) sea surface salinity (PSU).

In order to understand the periodicity of each abiotic factor, spectral analysis was applied to detrended time series. Significant Fourier frequencies at 1.0, corresponding to a quarterly rate, were observed in SST, PAR, mean atmospheric pressure and maximum tide level time series (Fig. 4A–D, respectively). Significant Fourier frequencies at 2.0, corresponding to a semestral rate, were observed in mean air humidity, minimum tide level and wind speed time series (Fig. 4E–G, respectively). A significant Fourier frequency at 1.3, corresponding to an approximate 4 months rate, was observed in SSS time series (Fig. 4H).

## 2 Experimental design, materials and methods

### 2.1 Data mining

Monthly abiotic data were collected from different online repositories, all regarding the period between January 2013 and December 2017, from localities near Mar Casado Beach rocky shore, in São Paulo State southern coast, Brazil. Different sources were used for the construction of this dataset as different monitoring initiatives presented specific monitoring resolutions, and there was no database that assembled all the abiotic data of interest for the same site.

Air temperature (°C), air humidity (%), atmospheric pressure (Hg mm) and dew point (°C) daily data were collected from WeatherUnderground [5] for Santos Air Base and missing points in time were filled with data from Instituto Nacional de Pesquisas Espaciais [6] for Itanhaém, São Paulo, Brazil. Data for SST (ºC) was extracted from the NOAA OI daily SST with a 0.25-degree resolution model [7] for the coordinates −23.875, −46.125. Monthly data for PAR (E/m^2^s = 1 mol of photons m^−2^ s^−1^ [SI]) was collected from NASA Ocean Color MODIS Terra satellite data bank [8] for the coordinates −23.979170, −46.187496. Monthly data for SSS (PSU = g/kg [SI]) were collected and assembled from NASA Aquarius [9] with a 1-degree resolution and Remote Sensing Systems [10] with a 0.25-degree resolution for the coordinates −24.5, −46.5 and −24.875, −46.625, respectively. Wind direction (º) in relation to the parallels and wind speed (Km/h) data were collected from Blended daily averaged 0.25-degree Sea Surface Winds assembly (at a 10 m level), provided by NOAA [11], for the coordinates −24.00, −45.75. Daily tidal level (m) data was collected from the Tide Board of Marinha do Brasil [12] for the Port of Santos. Averaging was applied to daily data in order to build a monthly database.

### 2.2 Principal component analysis

In order to identify co-variant abiotic variables, a Principal Component Analysis (PCA) was carried using the software R-0.3.5.0 [13]. Pre-treatment of the data before PCA was averaging the sampled months of January to March (Summer); April to June (Autumn); July to September (Winter) and October to December (Spring), centering (subtracting the mean value of each variable) and scaling (dividing each variable by its standard deviation) [14]. Afterwards, PCA was performed using the function *princomp* from the *stats* R package [13].

### 2.3 Time series analysis

A time series is a collection of observations indexed by time. The main features of a time series are trend and seasonal variations that can be modelled deterministically in function of time [15]. Each abiotic variable collected was treated as a continuous quarterly time series, using the average (as explained in section 2.2). While decomposing a time series, its trend and seasonal features might interact additively or multiplicatively. Once identified the relation of seasonal and trend variations, and log transforming the data if a multiplicative relation is observed, a linear regression (considering higher order polynomial in function of time and seasonal variations) was used to find a model that better fitted the observed data. Two models were considered: (I) linear model with seasonal variables:

**Table:** 

		β_1_, t = 1,5,9…
*x*_*t*_ = α_1_*t* + α_2_*t*^2^ + … + α_p_*t*^p^ + *s*_*t*_ + *z*_*t*_	*s*_*t*_=	β_2_, t = 2,6,10…
		β_3_, t = 3,7,11…
		β_4_, t = 4,8,12…

Where *x*_t_ is the modelled time series in function of time *t* (t = 1,2,…,20 [4]), α is a coefficient, *s*_*t*_ is the seasonal component for which each season has a coefficient *β* and *z*_*t*_ is the residual time series, defined as the difference between original data and fitted model; and (II) harmonic seasonal model:

*x*_*t*_ = α_0_ + α_1_*t* + α_2_*t*^2^ + … + α_p_*t*^p^ + (Σ_i=1_^[S/2]^ {*s*_*i*_ sin(2πi*t*/*S*) + *c*_*i*_ cos(2πi*t*/*S*)}) + *z*_*t*_

Where *x*_t_ is the modelled time series in function of time *t* (t = 1,2,…,20 [4]), α is a coefficient, *S* is the number of seasons (*S* = 4, for a quarterly time series), *s*_*i*_ and *c*_*i*_ are coefficients, and *z*_*t*_ is the residual time series, defined as above [15]. The best model was elected by the minimization of its Akaike Information Criterion (AIC), Bayesian Information Criterion (BIC) and Residuals Sums of Squares (RSS).

### 2.4 Trend analysis: structural breaks approach

A structural breaks approach to trend analysis is commonly used to identify intermediate time intervals in which the data follow an increasing or decreasing trend pattern that might be different from the global trend pattern [4]. The R package *strucchange* [16] includes functions that identify breakpoints in a deseasonalized time series. Deseasonalized time series were obtained by subtracting the seasonal component modelled in time series analysis (Section 2.3). The function *breakpoints* [16] was used to determine the number of structural breaks, using a maximum of 5 breaks (m = 5) and choosing the number of breaks which minimized the BIC and RSS. Finally, a linear regression was applied to each time interval, considering only first order polynomial in function of time (trend = α_0_ + α_1_*t*, t = 1,2,…,20 [4]).

### 2.5 Spectral analysis

Spectral analysis can be used to identify periodic events in a stationary time series that may be corrupted by other intrinsic variations [15]. Firstly, detrended time series were obtained by subtracting the trend component modelled in the time series analysis (Section 2.3). Time series stationarity was accessed using the function *adf.test* from the *tseries* R package [17]. When not stationary, differentiation was applied to the detrended time series imposing a differentiation order enough to make it stationary, using the function *diff* from the *base* R package [13]. The periodogram for each time series was then obtained using the function *spectrum* from the *stats* R package [13].
